# Influence of Higenamine on Exercise Performance of Recreational Female Athletes: A Randomized Double-Blinded Placebo-Controlled Trial

**DOI:** 10.3389/fpsyg.2021.633110

**Published:** 2021-09-07

**Authors:** Jelena S. Rasic, Nevena DJ. Ivanovic, Marija S. Andjelkovic, Ivana P. Nedeljkovic, Ivan R. Nikolic, Sava D. Stojanovic, Danijela K. Ristic-Medic, Marija M. Takic, Brizita I. Djordjevic, Nenad V. Dikic

**Affiliations:** ^1^Department of Bromatology, Faculty of Pharmacy, University of Belgrade, Belgrade, Serbia; ^2^Beo-lab Laboratories, Belgrade, Serbia; ^3^Sports Medicine Association of Serbia, Belgrade, Serbia; ^4^Faculty of Pharmacy, Singidunum University, Belgrade, Serbia; ^5^University Clinical Center of Serbia, Belgrade, Serbia; ^6^Institute for Medical Research, Centre of Research Excellence in Nutrition and Metabolism, Belgrade, Serbia; ^7^Faculty of Physical Education and Sports Management, Singidunum University, Belgrade, Serbia

**Keywords:** recreational athletes, female, ergogenic effects, safety assessment, higenamine, supplementation, dietary supplement

## Abstract

The aim of this study was to determine the ergogenic effects and the safety profile of a one-component higenamine supplement in female recreational athletes. Twelve recreational female basketball players (age 29–41 years, oxygen consumption (VO_2_max) > 30 ml⋅kg^–1^⋅min^–1^, with training > 5 h wk^–1^) were randomized either to the higenamine group, or to the placebo group for 3 weeks. In order to determine ergogenic effects and safety profile of higenamine administration, we assessed the following variables before and after 3 weeks of supplementation: anthropometric parameters, resting metabolic rate (RMR), exercise testing variables, serum free fatty acids (FFAs), blood pressure, enzyme activity, urea, lipid profile, and complete blood count. There were no differences between groups in anthropometric parameters, including basal metabolic rate (BMR), RMR and body fat [*p* = 0.706 (Cohen’s *d* 0.223), *p* = 0.169 (Cohen’s *d* 0.857), and *p* = 0.223 (Cohen’s *d* 0.750), respectively], FFAs [0.43 ± 0.03 vs. 0.54 ± 0.23, *p* = 0.206 (Cohen’s *d* 0.540)], neither significant differences in cardiopulmonary parameters after the intervention period. Furthermore, all measured outcome variables in the safety assessment were not significant, with values remaining stable during the intervention period for participants in both groups. This is the first study to document the effects and the safety profile of higenamine-based dietary supplements at a specified dose in female recreational athletes. Our data indicate that 21-day of supplementation with 75 mg higenamine would not result in improving cardiopulmonary exercise fitness and weight loss in female recreational athletes. Moreover, supplementation with 75 mg higenamine is safe and well-tolerated in younger recreational female athletes.

## Introduction

Higenamine could be found as an ingredient of fat-burn and pre-workout dietary supplements, which have become increasingly popular among competitive and recreational athletes due to its alleged ergogenic benefits (Cohen et al., 2019; Jagim et al., 2019; Yen et al., 2020). Higenamine, an alkaloid (6,7,12-trihydroxy-benzyl-1,2,3,4 tetrahydroisoquinoline), is found in many different plant species. In dietary supplements and over-the-counter products, it could be labeled in different names, such as norcoclaurine, demethylcoclaurine, or the name of the plant that is the source of higenamine: *Aconitum japonicum*, *Nandina domestica*, *Gnetum parvifolium*, *Asarum heterotropoides*, etc. (Okano et al., 2017; Grucza et al., 2018). As a non-selective beta-agonist, higenamine could show chronotropic and inotropic activity, has bronchodilatative effects, and enhances lipolysis (Stajic et al., 2017). As a β2-agonist, higenamine was added to the World Anti-doping Agency (WADA) prohibited list in 2017 under the S3 category, which mainly includes drugs for asthma treatment (WADA, 2017a; Yan et al., 2019; Feng et al., 2020). Higenamine was prohibited at all times, in and out-of-competition, which led to the fact that intentional and unintentional use of higenamine can pose a risk to athletes’ sporting careers (Grucza et al., 2018). Substances may be included in WADA prohibited list if they satisfy any two of the following three criteria; (1) it has the potential to enhance or enhances sport performance; (2) it represents an actual or potential health risk to the athlete; and (3) it violates the spirit of sport (Agency Wa-D., 2015). According to WADA’s report, positive cases of higenamine misuse have been increasing yearly. Moreover, higenamine came in second place among beta-2 agonists causing adverse analytical finding (WADA, 2017b). However, there is no strong evidence that higenamine has an ergogenic activity or an influence on bronchodilation. The previously conducted study did not fully address the underlying mechanisms of higenamine and therefore the efficacy and safety of oral higenamine is not full understood (Zhang et al., 2017). This is why anti-doping experts have raised concerns about fulfillment of the criteria for inclusion in the Prohibited List by higenamine (Stajic et al., 2017). Namely, according to Heuberger et al., only five out of 23 substance classes on the WADA prohibited list have robust evidence of actually having the ability to enhance sports performance in athletes (Heuberger and Cohen, 2019). Additionally there is still a lack of studies that would appropriately address the safety of the long-term use of higenamine-containing dietary supplements among recreational athletes, especially females. According to the previously observed findings the majority of users of fat-burn supplements were women between the ages of 18 and 34 who are more likely than males to experience the side effects of using this category of supplements (Jagim et al., 2019; Jędrejko et al., 2021).

Therefore, the aim of the study was to determine the ergogenic effects of a one-component higenamine supplement in recreational female basketball players during the 21-day administration period. Also, the study was conducted to examine for the first time the safety profile of chronic higenamine intake in female recreational athletes.

## Materials and Methods

### Experimental Approach to the Problem

In order to investigate the effects of long term higenamine supplementation, we assessed several variables prior and after the study: anthropometric parameters (fat and muscle content), resting metabolic rate (RMR), exercise testing variables [oxygen consumption (VO_2_max) and ventilation] and metabolic parameters [serum free fatty acid (FFA) oxidation]. In order to determine the safety profile of higenamine, blood pressure, enzyme activity, urea, creatinine, lipid profile, and complete blood count were analyzed. The supplementation lasted for 21 days, during which female basketball players were on the regular regimen of training. The experimental group received the higenamine capsules (25 mg) three times per day for 21 days following the instruction given by the manufacturer. Both groups had oral intake and were on the same dosing regimen of triple daily intake of active substance and placebo. The higenamine capsules purchased at a local pharmacy. The higenamine capsules were produced by Singular Sport (Higenamine by Singular Sport, United States) and quality and quantity control analysis was performed by local pharmaceutical company Essensa (Essensa, Belgrade, Serbia). The Essensa company produced the placebo capsules for study purposes that contained 100% erythritol. The higenamine capsules contained microcrystalline cellulose as excipient and both capsules were composed of hydroxypropylmethylcellulose (HPMC). In order to ensure high protocol adherence and promote participants’ commitment short message service (SMS) reminder system was established as well as communication via Viber group. At the final testing after the intervention, the participants were asked to return the remaining capsules.

The researchers counted the remaining capsules; the compliance in both groups was 90%. Both groups and the researchers were unaware of which group was receiving the higenamine until the statistical analyses were finished.

### Participants

The study included a randomized, double-blind, placebo-controlled group design. The recreational female basketball players were randomly allocated to the higenamine (*n* = 7) or the placebo group (*n* = 7). At the beginning of the study, two participants (one assigned to the placebo and the other to the experimental group) decided to cease participation due to personal reasons so the study involved 12 recreational female basketball players. Before participation, athletes completed medical history and physical activity questionnaires. Criteria for inclusion in the study: VO_2_max > 30 ml⋅kg^–1^⋅min^–1,^ with training > 5 h⋅wk^–1^. Exclusion criteria for diseases that could affect metabolism, such as: chronic diseases (neurological, renal, immune, etc.), cardiovascular diseases (high blood pressure, heart failure, cardiomyopathy, carditis, rheumatic heart disease, etc.), metabolic disorder (diabetes, malabsorption, cachexia, and acid–base imbalance), thyroid gland disease (hypothyroidism and hyperthyroidism), respiratory diseases (asthma), recent surgery, and acute infections.

To ensure compliance, athletes were asked to take the capsules before meals, the first one at 8 am, then at 1 pm and the last one at 6 pm. Furthermore, athletes were required to refrain from taking supplements and other medications and to hold steady training and regular diet regimen. A total of 12 recreational female basketball players completed the study. The physical and anthropometric characteristics of the athletes were similar (Table 1).

**TABLE 1:** Anthropometric characteristics of placebo and higenamine groups before and after the treatment.

							**Placebo vs. higenamine**	**Placebo vs. higenamine**
	**Placebo *n* = 6**		***p* Cohen’s *d***	**Higenamine *n* = 6**		***p* Cohen’s *d***	***p* (before) Cohen’s *d***	***p* (after) Cohen’s *d***
	**Before**	**After**		**Before**	**After**			
Age (years)	32.83 ± 5.15	/	/	33.00 ± 2.45	/	/	/	/
Height (m)	1.78 ± 0.09	/	/	1.77 ± 0.08	/	/	/	/
Weight (kg)	69.75 ± 11.46	69.87 ± 11.59	0.725	70.72 ± 5.50	71.08 ± 4.95	0.565	0.856	0.818
			0.162			0.248	0.108	0.136
BMI (kgm^–2^)	22.03 ± 2.08	21.98 ± 1.90	0.776	22.67 ± 1.87	22.73 ± 2.02	0.737	0.591	0.523
			0.122			0.131	0.324	0.382
BMR (kcal per day)	1486.17 ± 168.07	1491.17 ± 153.54	0.638	1488.67 ± 120.47	1463.00 ± 90.07	0.217	0.977	0.706*
			0.200			0.576	0.017	0.224
Body fat (%)	28.28 ± 5.45	28.25 ± 6.21	0.942	29.95 ± 5.06	32.05 ± 3.58	0.072	0.595	0.223*
			0.028			0.931	0.318	0.750
RMR (kcal per day)	1346.33 ± 289.04	1413.33 ± 251.59	0.518	1211.83 ± 413.96	1169.83 ± 313.48	0.683	0.529	0.169*
			0.284			0.177	0.377	0.857

*Results are expressed as mean ± standard deviations. Significance: *p* < 0.05 was obtained by paired *t*-test.*

*BMI, body mass index; BMR, basal metabolic rate; RMR, resting metabolic rate.*

*No significant difference in anthropometric parameters within study groups, including BMR (*p* = 0.706), RMR (*p* = 0.169), and body fat (*p* = 0.223).

All the experimental procedures in the current study followed the guidelines laid down in the Declaration of Helsinki. The Ethics Committee of the Sports Medicine Association of Serbia approved the study. Before enrolling in the study, all participants went through verbal and written consent processes.

### Dietary Intake and Physical Activity

Participants were instructed to maintain their habitual dietary intake throughout the study period. Dietary intake evaluation was based on participants’ subjective retrospective report, using the repeated 24-h recalls (24-h period prior to each test day) as the assessment method. Participants reported complete consumption of food and beverages conducted by a trained nutritionist in accordance with predefined, standardized protocol.

Participants were also instructed to maintain their usual physical activity patterns during the entire course of the study. Besides, they were required to report their training loads weekly, filling in the standard short form of International Physical Activity Questionnaire (IPAQ)^1^.

### Anthropometric Characteristics

Anthropometric characteristics were measured before and after the study. Body weight, body fat, basal metabolic rate (BMR), and body mass index (BMI) were determined using the bioimpedance method by body composition analyzer BC-418MA (Tanita, Tokyo, Japan).

Resting metabolic rate was measured by indirect calorimetry using desktop metabolic monitor Fitmate PRO (Fitmate, COSMED, Italy) based on the oxygen consumption (VO_2_) during rest.

### Blood Samples Collection and Analysis

Blood samples (3 mL per whole blood BD Vacutainer^®^ spray-coated K_2_EDTA tube and 10 ml per serum BD Vacutainer^®^ SST^TM^ II Advance tube) were taken out of the antecubital vein. Samples were collected twice: before and after the study, in the morning hours (between 7:00 am and 9:00 am), following 12 h overnight fasting in the absence of prior vigorous physical activity. Following collection, samples were processed, and fresh samples were analyzed for complete blood count on a fully automated hematology analyzer (Sysmex XT-1800i, Sysmex, Japan) that used fluorescence flow cytometry (FFC) technology: erythrocytes (RBC), hemoglobin (Hgb), hematocrit (Hct), platelets (PLT), leukocytes (WBC), and leukocyte formula. Blood samples were allowed to clot for 30 min at room temperature. Serum was separated using centrifugation 2,000 × *g*, 10 min by table top centrifuge (LACE32, COLO lab experts, Slovenia). One portion of serum was analyzed immediately; biochemical analysis, glucose, urea, creatinine, aspartate aminotransferase (AST), alanine aminotransferase (ALT), γ-glutamyl transferase (GGT), creatine kinase (CK), and lipids parameters were analyzed on the biochemical module Roche c501,which is an integral part of the integrated analyzer (Cobas^®^ 6000 Roche Diagnostics, Germany) by the method of spectrophotometric. One portion of the serum (3 mL) was frozen at −80°C until FFAs were analyzed. The FFAs were determined by the extraction of total lipids according to the method Folco using an internal standard 19–0. The separation of lipid classes in the amino propylene column (Agilent Technologies, United States), methylation, and were analyzed fatty acid methyl esters by gas chromatography (Shimadzu 2014, Japan).

### Cardiopulmonary Exercise Testing and Echocardiography Examination

Cardiopulmonary and echocardiography parameters were determined before higenamine supplementation and after 21 days of supplementation. Cardiopulmonary exercise testing (CPX) was performed on a treadmill (T-140, Cosmos, Germany), twice (before and after the study), according to maximal protocol. The exercise intensity was progressively increased, while oxygen and CO_2_ concentration of the inhaled and exhaled air were measured. A test was considered maximal if participants achieved 90% or more of predicted maximal heart rate (HR max) for age and sex, a plateau in VO_2_ was reached despite increased workload, a respiratory exchange ratio (RER) was greater than 1.00, and subjects reached volitional exhaustion. The maximal HR max was calculated as 220 minus the athlete age. Expiratory gases were collected on a breath-by-breath basis, and analyzed by metabolic cart (Quarck b2, COSMED, Italy). Ventilatory anaerobic threshold (VAT) was determined by the “V-slope” analysis of VO_2_ vs. carbon dioxide production (VCO_2_). The values of VO_2_ at Ventilatory Threshold [VT (VT I and VT II)] and at peak exercise (VO_2_ max) are expressed as ml O_2_kg^–1^min^–1^ during the 30 s in which the examined event occurred and printed using rolling averages every 10 s. The predicted peak of VO_2_ was defined according to current recommendations. The predicted peak O_2_ pulse was calculated as the predicted peak of VO_2_⋅ (predicted maximal HR) ^–1^. The RER 1.2 was considered as a satisfactory effort for athletes. Hemodynamic parameters obtained during the test were peak systolic blood pressure [SBP max (mmHg)], peak diastolic blood pressure [DBP max (mmHg)], HR max, as well as maximum voluntary ventilation (MVV), oxygen pulse. Criteria for test termination was determined by (a) VO2 plateau, (b) RER ≥ 1.2, (c) rating of perceived exertion ≥ 9/10, or maximal volitional exhaustion, and (d) HR above 90% of theoretical HR max (220 minus athlete age).

Standard 2D echocardiography was performed prior and after the study (Logiq 5, General Electric, United States). Athletes self-monitored blood pressure using device Blood Pressure Monitor (BP B1, Microlife, Microlife AG, Switzerland) every morning.

### Recording of Adverse Events

The adverse events were assessed by telephone interviews with participants each day during the 21-day intervention period, and they were recorded on the adverse event form throughout the study. The participants were asked to self-report any health-related problems or symptoms they were experiencing during the intervention period.

### Statistical Analysis

Numerical data were presented by arithmetic mean and standard deviation. Normal distribution was evaluated using mathematical (coefficient of variation, skewness, kurtosis, Kolmogorov–Smirnov test, and Shapiro-Wilk test) and graphical methods (histogram, Q–Q diagram, and a box plot). Interventional and control groups were compared with Student *t*-test, and values before and after in each group by *t*-test for dependent samples. As a measure of effect size, Cohen’s *d* was used for paired or independent *t*-tests where appropriate. For exploring associations between parameters, we used the Pearson linear correlation coefficient. The results were expressed as mean value and standard deviation. All statistical methods were considered significant for the level of 0.05. The analysis was realized in software IBM SPSS version 21 (IBM Corp. Released 2012. IBM SPSS Statistics for Windows, Version 21.0. Armonk, NY: IBM Corp.). As the conclusion according to the test statistic will be the same as according to the previously reported *p*-value for the chosen error of the first type α = 0.05, we will report *p*-value with the effect size expressed by Cohen’s *d* for student *t*-test for two independent as well as for paired samples where appropriate. Those values are reported in all the tables. G power software ver. 3.1.9.2 was used to perform power analysis calculations.

## Results

A total of 12 female recreational basketball players with mean age of 33.00 ± 2.45 years were recruited. They were compared within two groups: Higenamine (Experimental) and Placebo before and after the intervention period of 21 days, as well as between the groups.

### Anthropometric Parameters

There was no significant change in anthropometric parameters within the study groups, including BMR, RMR and body fat [*p* = 0.706 (Cohen’s *d* 0.223), *p* = 0.169 (Cohen’s *d* 0.857), *p* = 0.223 (Cohen’s *d* 0.750), respectively] (Table 1).

### Biochemical and Metabolic Parameters

According to blood parameters, there was a significant change in MCV and MCHC in placebo group before and after the intervention period (*p* = 0.048 and 0.049, respectively). There were no differences in any blood count parameters between the groups before and after the intervention period (Table 2). Moreover, the athletes did not differ in any of the analyzed metabolic parameters, except blood glucose concentration in the placebo group before and after the study finished (*p* = 0.035) (Table 3).

**TABLE 2:** Blood parameters in placebo and higenamine groups before and after the treatment.

	**Placebo *n* = 6**			**Higenamine *n* = 6**			**Placebo vs. higenamine**	**Placebo vs. higenamine**
	**Before**	**after**	***P* Cohen’s *d***	**before**	**After**	***p* Cohen’s *d***	***p* (before) Cohen’s *d***	***p* (after) Cohen’s *d***
WBC (10^9^ L^–1^)	5.89 ± 1.27	6.97 ± 1.09	0.094	6.90 ± 2.14	6.46 ± 2.42	0.338	0.345	0.649
			0.847			0.403	0.574	0.272
RBC (10^12^ L^–1^)	4.33 ± 0.55	4.40 ± 0.48	0.344	4.48 ± 0.18	4.53 ± 0.28	0.651	0.552	0.556
			0.458			0.181	0.367	0.331
Hgb (gL^–1^)	124.50 ± 11.26	127.17 ± 7.91	0.175	129.50 ± 5.92	131.83 ± 8.08	0.446	0.358	0.336
			0.646			0.337	0.556	0.583
Hct (LL^–1^)	0.38 ± 0.04	0.38 ± 0.03	0.453	0.39 ± 0.02	0.39 ± 0.02	0.541	0.473	0.390
			0.000			0.000	0.316	0.333
MCV (fL)	87.05 ± 5.60	86.78 ± 5.50	0.131	86.52 ± 0.86	86.77 ± 1.56	0.498	0.826	0.994
			0.740			0.298	0.132	0.002
MCH (pg)	28.97 ± 2.86	29.15 ± 2.96	0.048*	28.90 ± 0.35	29.08 ± 0.30	0.325	0.957	0.957
			1.097			0.438	0.034	0.033
MCHC (gL^–1^)	332.50 ± 17.76	335.33 ± 19.46	0.047*	334.17 ± 4.45	335.33 ± 4.68	0.528	0.828	1.000
			1.067			0.275	0.12	0.000
PLT (10^9^ L^–1^)	225.83 ± 19.55	234.17 ± 18.27	0.152	253.50 ± 64.38	251.00 ± 63.74	0.828	0.338	0.548
			0.690			0.094	0.582	0.359
Neutrophils (%)	50.93 ± 8.89	57.47 ± 10.63	0.145	51.55 ± 7.96	41.35 ± 4.48	0.928	0.902	0.223
			0.705			1.978	0.073	1.976
Lymphocytes (%)	37.32 ± 9.28	31.80 ± 10.91	0.148	37.83 ± 7.46	37.10 ± 3.60	0.756	0.917	0.285
			0.699			0.134	0.061	0.652
Monocytes (%)	8.65 ± 1.49	8.20 ± 0.87	0.297	7.35 ± 1.71	7.75 ± 1.82	0.453	0.190	0.597
			0.474			0.332	0.811	0.315
Eosinophils (%)	2.45 ± 0.57	1.95 ± 0.65	0.070	2.63 ± 1.98	3.10 ± 2.31	0.060	0.831	0.286
			0.934			1.005	0.124	0.678
Basophils (%)	0.65 ± 0.36	0.58 ± 0.23	0.675	0.63 ± 0.19	0.70 ± 0.11	0.328	0.922	0.291
			0.192			0.455	0.069	0.666
Neutrophils (10^9^ L^–1^)	3.02 ± 0.85	4.01 ± 0.99	0.095	3.49 ± 0.84	3.29 ± 1.13	0.588	0.358	0.268
			0.841			0.238	0.556	0.678
Lymphocytes (10^9^ L^–1^)	2.19 ± 0.73	2.21 ± 0.86	0.861	2.66 ± 1.15	2.39 ± 0.89	0.142	0.424	0.735
			0.069			0.718	0.488	0.206
Monocytes (10^9^ L^–1^)	0.50 ± 0.11	0.57 ± 0.11	0.099	0.51 ± 0.20	0.51 ± 0.24	0.920	0.943	0.548
			0.855			0.000	0.062	0.321
Eosinophils (10^9^ L^–1^)	0.14 ± 0.05	0.13 ± 0.04	0.723	0.20 ± 0.22	0.23 ± 0.27	0.276	0.500	0.399
			0.285			0.520	0.376	0.518
Basophils (10^9^ L^–1^)	0.04 ± 0.02	0.04 ± 0.02	0.415	0.05 ± 0.02	0.05 ± 0.02	1.000	0.501	0.755
			0.000			0.000	0.500	0.500

*Results are expressed as mean ± standard deviations. Significance was considered for *p* < 0.05.*

*WBC, white blood cell; RBC, red blood cell; MCV, mean cell volume; MCH, mean cell hemoglobin; MCHC, mean cell hemoglobin concentration.*

*There was a significant difference in MCV and MCHC in placebo group before and after the intervention period (*p* = 0.048 and 0.049). There were no significant differences in any blood count parameters between the groups before and after the intervention period.

**TABLE 3:** Metabolic parameters in placebo and higenamine groups before and after the treatment.

							**Placebo vs. higenamine**	**Placebo vs. higenamine**
	**Placebo *n* = 6**		***p* Cohen’s *d***	**Higenamine *n* = 6**		***p* Cohen’s *d***	***p* (before) Cohen’s *d***	***p* (after) Cohen’s *d***
	**Before**	**After**		**Before**	**After**			
Glucose (mmol⋅L^–1^)	4.52 ± 0.32	4.98 ± 0.41	0.035*	4.45 ± 0.42	4.73 ± 0.24	0.108	0.764	0.229
			1.158			0.798	0.187	0.744
Urea (mmol⋅L^–1^)	4.65 ± 0.80	4.32 ± 0.82	0.472	3.87 ± 0.87	3.90 ± 0.81	0.921	0.135	0.396
			0.314			0.038	0.933	0.515
Creatinine (μmol⋅L^–1^)	83.67 ± 11.36	78.50 ± 10.60	0.141	76.33 ± 9.56	75.00 ± 10.84	0.444	0.254	0.584
			0.713			0.338	0.699	0.326
AST (IU⋅L^–1^)	15.83 ± 2.64	16.17 ± 3.37	0.611	16.67 ± 4.97	15.50 ± 1.97	0.420	0.724	0.685
			0.226			0.359	0.211	0.243
ALT (IU⋅L^–1^)	11.67 ± 3.01	12.17 ± 2.86	0.296	11.17 ± 3.82	12.00 ± 3.29	0.289	0.806	0.927
			0.475			0.482	0.145	0.055
GGT (IU⋅L^–1^)	10.50 ± 2.51	10.83 ± 2.71	0.465	14.00 ± 3.16	14.33 ± 3.08	0.465	0.060	0.063
			0.320			0.321	1.227	1.207
CK (IU⋅L^–1^)	89.00 ± 41.02	106.17 ± 58.10	0.139	126.00 ± 140.29	106.17 ± 58.69	0.601	0.549	1.000
			0.717			0.228	0.358	0.000
Cholesterol (mmol⋅L^–1^)	3.91 ± 0.82	3.93 ± 0.72	0.863	4.26 ± 0.64	4.30 ± 0.74	0.827	0.431	0.406
			0.075			0.107	0.476	0.507
HDL-C (mmol⋅L^–1^)	1.58 ± 0.19	1.59 ± 0.22	0.894	1.35 ± 0.33	1.37 ± 0.29	0.548	0.169	0.163
			0.057			0.293	0.854	0.855
LDL-C (mmol⋅L^–1^)	2.04 ± 0.65	2.01 ± 0.55	0.697	2.50 ± 0.76	2.57 ± 0.77	0.624	0.282	0.172
			0.165			0.209	0.651	0.837
Triglycerides (mmol⋅L^–1^)	0.66 ± 0.07	0.74 ± 0.19	0.327	0.91 ± 0.33	0.80 ± 0.27	0.303	0.112	0.691
			0.412			0.433	1.048	0.257
FFA (mmol⋅L^–1^)	0.43 ± 0.03	0.54 ± 0.23	0.206	0.48 ± 0.16	0.53 ± 0.25	0.537	0.475†	0.896†
			0.540			0.278	0.434	0.042

*Results are expressed as mean ± standard deviations. Significance was considered for *p* < 0.05.*

*GGT, γ-glutamyl transferase; AST, aspartate aminotransferase; ALT, alanine aminotransferase; CK, creatine kinase; HDL-C, high-density lipoprotein cholesterol; LDL-C, low-density lipoprotein cholesterol. FFA, free fatty acids.*

*There was a significant difference in blood glucose concentration in the placebo group before and after the study finished (*p* = 0.035). There were no significant differences in any metabolic parameters between the groups before and after the intervention period.

*^†^There was no significant difference in free fatty acids (FFA) values between in the placebo and the higenamine group before the intervention (*p* = 0.475), neither after the study finished (*p* = 0.896).*

### Dietary Intake and Physical Activity

According to the IPAQ participant’s average calorie consumption in average per week was between 1,820 and 2,650 kcal⋅week^–1^, which classified them in the group of high physical activity level. Participants maintained their usual physical activity patterns during the entire course of the study as it was requested from the participants. Food consumption analyses, measured by 24-h recall repeated on random days, confirmed stable dietary intake during the experimental period, since no significant differences were observed in energy intake.

### Free Fatty Acid

Results of FFAs are presented in Table 3. FFAs did not change significantly during the intervention period in the placebo group [0.43 ± 0.03 vs. 0.54 ± 0.23, *p* = 0.206 (Cohen’s *d* 0.540)], neither in the higenamine group [0.48 ± 0.16 vs. 0.53 ± 0.25, *p* = 0.537 (Cohen’s *d* 0.278)]. There was no difference in FFAs values between the placebo and the higenamine group before the intervention, neither after the study finished [*p* = 0.475 (Cohen’s *d* 0.434), *p* = 0.896 (Cohen’s *d* 0.042), respectively].

### Cardiopulmonary and Echocardiography Parameters

The only significant difference noticed after the 21-day intervention period was the slightly lower value of RER within the higenamine group after the supplementation [1.08 ± 0.07 vs. 1.05 ± 0.07, *p* = 0.020 (Cohen’s *d* 1.154)]. Still, there was no significant difference between the placebo and the higenamine group (1.05 ± 0.06 vs. 1.05 ± 0.07, *p* = 0.931). There were no significant differences in cardiopulmonary parameters: maximum oxygen consumption (VO_2_ max), maximum ventilation (VE max), HR max, recovery of HR in the first, second, or third minute, as well as ejection fraction (EF), mitral inflow (E/A) on echocardiography. All measured cardiopulmonary and echocardiography parameters are presented in Table 4.

**TABLE 4:** Cardiopulmonary and echocardiography parameters in placebo and higenamine groups before and after the treatment.

							**Placebo vs. higenamine**	**Placebo vs. higenamine**
	**Placebo *n* = 6**		***p* Cohen’s *d***	**Higenamine *n* = 6**		***p* Cohen’s *d***	***p* (before) Cohen’s *d***	***p* (after) Cohen’s *d***
	**Before**	**After**		**Before**	**After**			
**Echocardiography parameters**								
EF (%)	67.50 ± 4.76	67.67 ± 3.88	0.908	69.00 ± 2.53	70.00 ± 0.00	0.377	0.511	0.201
			0.050			0.395	0.394	0.849
E (VP m⋅ s^–1^)	0.93 ± 0.18	0.92 ± 0.15	0.919	0.99 ± 0.15	0.93 ± 0.10	0.318	0.501	0.912
			0.259			0.463	0.362	0.078
A (VP m⋅ s^–1^)	0.75 ± 0.18	0.73 ± 0.17	0.636	0.67 ± 0.06	0.64 ± 0.13	0.505	0.369	0.336
			0.276			0.276	0.596	0.595
**Cardiopulmonary parameters**								
VO_2_ max (ml⋅kg^–1^⋅min^–1^)	37.32 ± 7.66	39.58 ± 7.00	0.285	38.20 ± 6.39	36.92 ± 4.36	0.296	0.833	0.447
			0.488			0.474	0.125	0.456
VO_2_ max (ml⋅min^–1^)	2587.17 ± 626.27	2763.82 ± 603.49	0.223	2707.40 ± 557.99	2624.38 ± 371.27	0.399	0.733	0.640
			0.569			0.377	0.203	0.278
VE max (L⋅min^–1^)	84.33 ± 22.90	87.78 ± 21.40	0.493	88.35 ± 16.94	83.57 ± 18.07	0.249	0.737	0.720
			0.302			0.534	0.200	0.213
SBP max (mmHg)	158.33 ± 26.39	158.33 ± 14.72	1.000	160.00 ± 8.94	165.00 ± 12.25	0.203	0.886	0.414
			0.000			0.597	0.085	0.493
DBP max (mmHg)	66.67 ± 8.16	65.00 ± 8.37	0.363	61.67 ± 4.08	61.67 ± 4.08	1.000	0.209	0.401
			0.409			0.000	0.775	0.506
HR on VT II (bpm⋅min^–1^)	166.00 ± 7.35	168.83 ± 11.48	0.480	164.67 ± 6.92	158.00 ± 17.26	0.231	0.753	0.229
			0.311			0.557	0.186	0.739
HR on VT II (%)	92.05 ± 4.16	93.13 ± 3.09	0.662	93.77 ± 4.15	90.25 ± 11.11	0.297	0.491	0.554
			0.189			0.475	0.414	0.353
HR on VT I (bpm⋅min^–1^)	148.33 ± 24.09	149.67 ± 16.88	0.702	142.50 ± 8.83	148.50 ± 4.97	0.204	0.590	0.874
			0.166			0.596	0.321	0.094
HR on VT I (%)	81.72 ± 8.47	82.45 ± 6.04	0.601	81.20 ± 5.61	84.67 ± 2.41	0.144	0.903	0.423
			0.227			0.707	0.072	0.483
HR max (bpm⋅min^–1^)	180.67 ± 10.63	181.17 ± 8.33	0.765	175.67 ± 5.82	175.50 ± 7.31	0.889	0.336	0.239
			0.129			0.061	0.583	0.724
HR max (%)	94.58 ± 8.31	94.85 ± 7.66	0.768	93.90 ± 3.84	93.80 ± 4.36	0.876	0.859	0.776
			0.129			0.067	0.105	0.168
HR rest 1 min (bpm⋅min^–1^)	153.17 ± 13.20	151.50 ± 8.94	0.575	149.67 ± 6.19	146.67 ± 9.67	0.256	0.569	0.390
			0.246			0.524	0.340	0.519
HR rest 2 min (bpm⋅min^–1^)	130.67 ± 13.72	126.67 ± 11.99	0.430	129.67 ± 7.99	125.17 ± 12.77	0.178	0.880	0.838
			0.285			0.562	0.089	0.121
HR rest 3 min (bpm⋅min^–1^)	118.00 ± 13.55	117.00 ± 10.00	0.835	117.17 ± 12.12	112.67 ± 13.59	0.093	0.913	0.543
			0.090			0.848	0.065	0.363
MVV (L⋅min^–1^)	151.67 ± 17.29	151.67 ± 17.29	1.000	151.58 ± 15.13	151.57 ± 15.12	0.363	0.993	0.992
			0.000			0.001	0.006	0.006
O_2_ (HR) ^–1^ (ml⋅bpm^–1^)	14.33 ± 3.50	15.08 ± 3.23	0.287	15.75 ± 3.40	14.33 ± 1.97	0.129	0.494	0.638
			0.238			0.525	0.412	0.280
VE/VCO_2_ slope	28.65 ± 3.56	30.23 ± 2.68	0.256	27.10 ± 5.78	27.83 ± 2.27	0.721	0.588	0.125
			0.523			0.147	0.323	0.966
RER	1.06 ± 0.07	1.05 ± 0.06	0.537	1.08 ± 0.07	1.05 ± 0.07	0.020*	0.597	0.931*
			0.236			1.154	0.286	0.000
Test duration (min)	8.52 ± 2.99	9.17 ± 2.30	0.204	7.87 ± 2.64	9.35 ± 3.18	0.063	0.698	0.915
			0.592			0.971	0.230	0.065

*Results are expressed as mean ± standard deviations. Significance was considered for *p* < 0.05.*

*EF, ejection fraction; E/A, mitral inflow; VO_2_ max, maximum oxygen consumption; VE max, maximum ventilation; SBP max, maximum systolic blood pressure; DBP max, maximum diastolic blood pressure; HR max, maximum heart rate; VT I, first ventilatory threshold; VT II, second ventilatory threshold; MVV, maximum voluntary ventilation; O_2_ (HR) ^–1^, oxygen pulse; RER, respiratory exchange ratio; bpm (beat per minute).*

*A significant difference noticed after the 21-day intervention period was the slightly lower value of RER within higenamine group after the supplementation (*p* = 0.020). There was no significant difference between the placebo and the higenamine group (*p* = 0.931). There were no significant differences in cardiopulmonary parameters: VO_2_ max, maximum ventilation (VE max), HR max, recovery of HR in the first, second, or third minute, as well as ejection fraction (EF), mitral inflow (E/A) on echocardiography.

During the 21-day intervention, there were no statistically significant changes of systolic or DBP between the higenamine and the placebo groups, neither within groups.

### Adverse Effects

Adverse events were self-reported by the participants regardless of suspected causal relationship to the study treatments. Athletes from both groups, the placebo, and the higenamine group, reported adverse events. A total of 23 side effects were reported during the intervention period from participants in both groups. Two of the six athletes from the placebo group experienced a headache at the beginning of the study. In the higenamine group four of the six athletes also self-reported adverse events such as headache and one reported dry mouth besides headache: the first participant experienced headache on the 1st and 7th day of the intervention; the second participant experienced headache on the 2nd, 3rd, and 4th day; the third participant reported headache on the 9th and 13th day and the fourth participant reported dry mouth on the 2nd and 3rd day. All the reported adverse events were transient. No other adverse events were noted for any participants.

## Discussion

Findings from this study indicate that 21-day supplementation with higenamine does not result in a statistically significant change in any of the measured outcome variables. These data are specific to a sample of healthy, young female recreational athletes consuming daily 75 mg of higenamine with respect to the chosen outcomes measures only. According to our data, supplementation with 75 mg higenamine is safe and well-tolerated in younger recreational female athletes (Jagim et al., 2019).

The characteristics of the athletes enrolled in the study were very similar, and there were no differences between the groups (Table 1). Participants were taking 75 mg of higenamine daily divided into three doses, following the instructions for use given by the supplement manufacturer. According to the previously observed findings, this dose of higenamine is a standard dose found in dietary supplements. Namely, Cohen et al. analyzed dietary supplements available for sale in the United States for the presence and quantity of higenamine and reported that average dosages of 62 ± 6.0 mg per serving of higenamine were found in dietary supplements. Moreover, the same researchers have shown that the quantity of higenamine in supplements can vary from 0.01 to 200% relative to the declared dose (Cohen et al., 2019). Therefore, in this study, the declared amount of 75 mg, which corresponds to the dose to which consumers are most often exposed in dietary supplements, was analytically confirmed before the beginning of the study.

After the 21-day intervention period, there were no differences between and within the groups, in the measured value of RMR, neither difference in bodyweight and total body fat (%) (Table 1). Moreover, after the 21-days of treatment, there was no increase in the mobilization of fatty acids, which was assessed by measuring fasting concentrations of fatty acids (Table 3). Data in the literature have shown that beta_2_-agonists has the potential to enhance performance, lipolysis, and increase thermogenesis (Riiser et al., 2020). Furthermore, as a non-selective beta-agonist, higenamine could also have agonistic activity on beta_3_-receptors mainly expressed in white adipose tissue, which could also result in fat loss by stimulating fatty acid mobilization and, directly or indirectly, fat oxidation (Hostrup et al., 2020). However, a limited number of human studies have examined lipolytic and thermogenic effects of higenamine. The only published human study determining the impact of a higenamine-based supplement on measures of lipolysis and metabolic rate was the study conducted by Lee et al. (2013). In this study, the authors examined the acute effect of a supplement that contained besides higenamine, also Yohimbe bark extract and caffeine, both stimulants. Acute oral administration of this higenamine-based supplement led to an increase in energy expenditure and circulating fatty acids, including an increase in blood pressure and HR in the group of healthy, active, and young male and female participants. Since each of these three components could exert these effects according to their mechanism of action, the authors were not able to conclude whether higenamine influenced the measured outcomes. In another study conducted by the same group of authors, in which one group of the male recreational participants had been taking higenamine during 8 weeks, only safety profile of higenamine supplements was examined (Bloomer et al., 2015). On the contrary, in our study the effects were monitored both before the start of the study and after the end of the study, at least 12 h after the ingestion of the last capsule so we did not measure acute effects as it was performed in the study conducted by Lee et al. (2013). Considering that long-term increases in energy expenditure and lipolysis would most likely lead to changes in body weight and body composition it would be realistic to expect any significant change in the higenamine group after 21 days of treatment. However, no significant differences were found between the groups in any measured parameters that could indicate an increase in energy expenditure, weight loss, or fat oxidation, including lipid status parameters.

As it is presented in Table 4, there were no differences in almost any of the cardiopulmonary parameters between groups, neither within the higenamine group, after the intervention period. The only statistically significant change noticed after the 21-days intervention period was the slightly lower value of RER within higenamine group after the treatment although no statistical significance was reached between the groups. The RER [(CO_2_ production⋅ (O_2_ uptake) ^–1^] increase with the exercise intensity and measured under steady-state is commonly used to indirectly determine the relative contribution of carbohydrate and lipids to overall expenditure, whereas high RER suggests carbohydrate oxidation and low RER predominantly lipid oxidation. The RER is affected by factors such as diet and previous exercise, and a sedentary lifestyle increases the RER shifting metabolism from lipid to predominantly carbohydrate oxidation and fat deposition (Ramos-Jiménez et al., 2008). Higenamine supplementation resulted in a subtle decrease in RER in female participants. Still, this change did not result in an increase in serum-FFAs, fat mass, or in any other related parameter.

*In vivo* and *in vitro* studies showed that higenamine could exert positive inotropic and chronotropic effects, as well as bronchodilator effects, as a result of its beta_2_-agonistic activity (Zhang et al., 2017). These effects were also demonstrated in human studies, which included healthy volunteers or patients with heart disease or suspected heart disease. However, in all these studies reporting consistently increased HR and variable effects on blood pressure followed by dyspnea, dizziness, and other adverse effects, higenamine was administered intravenously, mostly using gradual infusions in doses of 2.5 or 5 mg higenamine (Cohen et al., 2019). Furthermore, the bronchodilator effects of higenamine were only demonstrated in *in vitro* studies (Kato et al., 2017; Zhang et al., 2017), and the safety profiles of higenamine are based on a small population of patients (Zhang et al., 2017).

While the pharmacokinetics of higenamine after intravenous administration has been intensively studied in animals and humans, the data are scarce regarding the oral route of administration in humans. The only data in the literature are those obtained in the rabbit and rat model showing very poor bioavailability of higenamine (Lo and Chen, 1996; Wang et al., 2020). Moreover, there are no studies providing data that would allow comparison of intravenous and oral doses of higenamine. Therefore, it is still unknown what doses of higenamine taken orally can lead to the effects on the cardiorespiratory system observed after intravenous administration *in vivo* and *in vitro* studies. However, the results of our study indicate that 75 mg of higenamine taken during 21 days would not cause any cardiopulmonary effects.

After intravenous administration, higenamine has a very short half-life, almost ninety-four percent of higenamine can be eliminated from the body within 30 min (Feng et al., 2012). This could lead to the conclusion that no physiological effect can be detected 12 h after the last dose taken. However, the group of authors who measured urine concentration after 7 days of repeated *Plumula nelumbinis* capsule administration, showed that even 3 days after drug withdrawal, higenamine could be detected in urine at concentrations ≥ 10 ng/mL which is WADA reporting limit (Yan et al., 2019). These data strongly indicate differences in the pharmacokinetics of higenamine following oral and intravenous administration and thus could explain the difference in physiological effect in two routes of administration.

In order to assess the safety of chronic administration of higenamine at a dose of 75 mg, complete blood counts, enzymes (CK, AST, and ALT), urea, creatine, lipid parameters, and blood pressure measures were taken into account. In the safety assessment, cardiopulmonary parameters were also taken into consideration, and adverse events were recorded during and 7 days after the intervention period.

All measured outcome variables in this study were not significant, with values remaining stable during the intervention period for participants in both groups (Tables 2, 3). No changes in blood pressure were observed between the groups as well as within the higenamine group. The only adverse events that were reported by female recreational athletes were headache occurred in participants from both (placebo and intervention) groups, and dry mouth, which was reported only by one participant in higenamine groups. Both of these adverse events were self-reported in the first days of the intervention. Data in the literature have shown that intravenous administration of higenamine in healthy volunteers could be associated with the occurrence of headache and dry mouth (Zhang et al., 2017; Cohen et al., 2019). In our study such effects were reported with oral administration of higenamine, although headache was reported in the placebo group, in addition to the higenamine group In the above mentioned study, in which the safety of higenamine supplementation was monitored in doses of approximately 100 mg for 8 weeks in male healthy recreational participants, there are no reported changes in measured cardiopulmonary parameters, neither any adverse events (Bloomer et al., 2015). One case report of the adverse effect of higenamine in young men who developed rhabdomyolysis after consuming a higenamine-based supplement and completed intensive exercise routine was published in the literature (Jeter et al., 2015). However, the case report did not provide any information on the amount of higenamine and other ingredients present in the supplement.

Although the underlying mechanisms of higenamine have not been fully understood and data. of both safety/unsafety and ergogenic effects of orally administrated higenamine is still lacking, higenamine was added to the WADA prohibited list, and ever since 2017 higenamine-containing supplements and products can pose an immediate risk to competitive athletes’ careers.

The key limitation of the present study is a small sample of recreational athletes that potentially decreased the chance of finding significant differences in measured clinical outcomes. However, as it is easy to see in the results, there are only a few parameters with large effect size, but this difference is not clinically important, so it allows us to conclude that all insignificant *p*-values are representative for this experimental situation. The sample size of 6 respondents per group was enough for some outcomes, like body fat (%) and HR rest, but insufficient for some other. That is why further investigations with larger sample size groups are needed. According to the chosen error of the first type, total number of respondents of 12, and achieved effect size (Cohen’s *d*) of 0.931 for body fat (%), and of 0.848 for HR after 3 min of rest, the study power was 83.50 and 76.25%, respectively. Furthermore, in order to extend our findings, additional study involving the use of different dosages of higenamine is needed, However, the advantage of the study is the vast array of parameters that have been measured in order to address the efficacy and safety of the long-term use of higenamine-containing dietary supplements. In addition, this is the first study to document the effects and safety profile of higenamine-based dietary supplements at a specified dose in female recreational athletes.

In conclusion, 21-day supplementation with 75 mg higenamine does not result in changing of cardiopulmonary exercise fitness nor in the effect of weight loss in female recreational athletes. Moreover, our data indicate that supplementation with 75 mg higenamine is safe and well-tolerated in younger female recreational athletes.

## Conclusion

The current study demonstrates the safety of oral use of higenamine at 75 mg doses for 3 weeks in female recreational athletes. Another important aspect of this study is that these results indicate that daily intake of 75 mg higenamine during 3 weeks would not result in improving cardiopulmonary exercise fitness or weight loss. All of the above leads to the conclusion that higenamine under these conditions of application does not exhibit beta-agonistic activity, which was the criteria for adding higenamine on the WADA Prohibited List.

## Data Availability Statement

The raw data supporting the conclusions of this article will be made available by the authors, without undue reservation.

## Ethics Statement

The Ethics Committee of the Sports Medicine Association of Serbia approved the study. The patients/participants provided their written informed consent to participate in this study.

## Author Contributions

JR, MA, BD, and ND were involved in conception, design, and coordination of the study. JR, IPN, IRN, and SS were involved in the subject recruitment and data collection. JR, NI, MA, DR, MT, BD, and ND were involved in the data interpretation, statistical analysis, and writing the manuscript. All authors critically reviewed the manuscript, have read and approved the publication of this final version of the manuscript, contributed to the article, and approved the submitted version.

## Conflict of Interest

The authors declare that the research was conducted in the absence of any commercial or financial relationships that could be construed as a potential conflict of interest.

## Publisher’s Note

All claims expressed in this article are solely those of the authors and do not necessarily represent those of their affiliated organizations, or those of the publisher, the editors and the reviewers. Any product that may be evaluated in this article, or claim that may be made by its manufacturer, is not guaranteed or endorsed by the publisher.

## Footnotes

1
http://www.ipaq.ki.se/downloads.htm
